# Contrast-harmonic endoscopic ultrasound-assisted endoscopic ultrasound-guided hepaticogastrostomy for minimally dilated intrahepatic ducts

**DOI:** 10.1055/a-2908-4930

**Published:** 2026-07-31

**Authors:** Shan-Shan Hu, Wei-Hui Liu

**Affiliations:** 1 Department of Gastroenterology and Hepatology 89669 Sichuan Provincial People’s Hospital, School of Medicine, University of Electronic Science and Technology of ChinaChengduSichuan ProvinceChina

Minimally dilated intrahepatic bile ducts (<5 mm) pose significant technical challenges in endoscopic ultrasound (EUS)-guided hepaticogastrostomy (EUS-HGS) due to their sonographic similarity to adjacent portal venous branches. Color Doppler imaging is frequently compromised by cardiac motion artifacts and angle dependence, precluding reliable real-time guidance. Contrast-harmonic EUS (CH-EUS) overcomes these fundamental limitations by detecting microbubble harmonic signals at frequencies distinct from tissue motion artifacts, thereby enabling reliable real-time differentiation irrespective of cardiac or respiratory cycles. 1​ 2​ ​3​ We recently encountered a technically demanding case in which CH-EUS successfully delineated the anatomical relationship between a diminutive intrahepatic bile duct (3–4 mm) and the adjacent portal vein, underscoring its unique value in anatomically challenging scenarios.

**Fig. 1:**
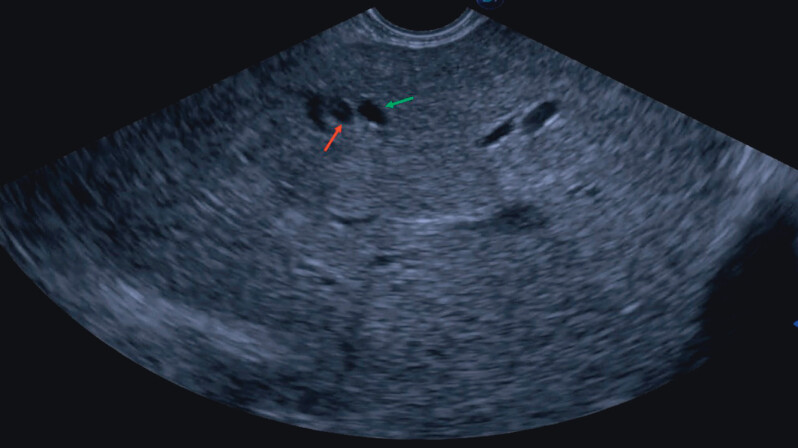
Gray-scale EUS: a minimally dilated intrahepatic bile duct (green arrow) and a portal vein (red arrow).

**Fig. 2:**
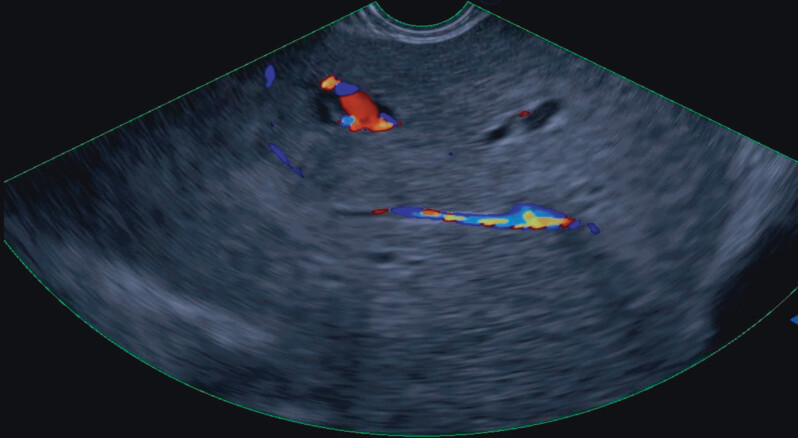
Color Doppler: rhythmic flickering with cardiac motion artifacts.

**Fig. 3:**
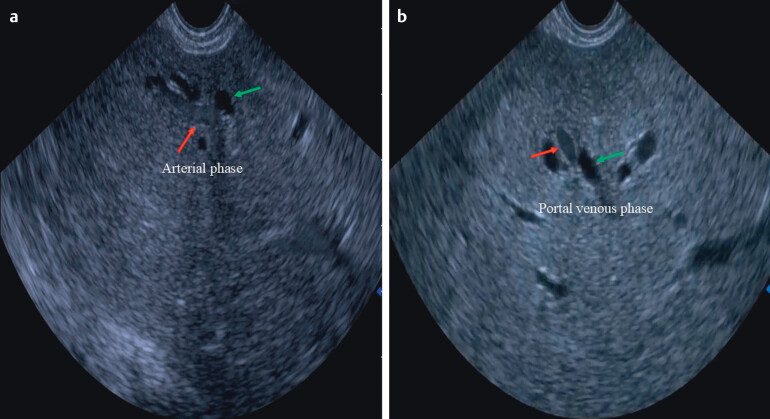
CH-EUS differential enhancement. ( **a** ) Arterial phase: Hepatic artery shows hyperenhancement (red arrow), and the bile duct is avascular (green arrow). ( **b** ) Portal venous phase: The portal vein has bright enhancement (red arrow), and the bile duct is non-enhanced (green arrow).

**Fig. 4:**
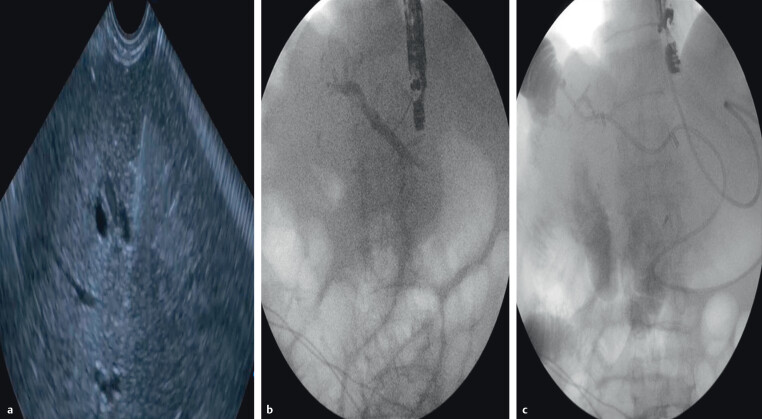
Real-time CH-EUS guidance. ( **a** ) Avascular needle trajectory planning and 19-gauge needle cannulation. ( **b** ) Cholangiography confirming the position. ( **c** ) 7Fr nasobiliary drain deployment.

A 68-year-old man presented with obstructive jaundice secondary to a hepaticojejunostomy stricture following previous hepatocellular carcinoma resection. EUS revealed a 3-mm left intrahepatic duct inseparable from the adjacent portal vein branch on conventional gray-scale imaging ( Fig. 1 ). Color Doppler demonstrated portal venous flow with rhythmic flickering synchronized with cardiac motion, compromising continuous visualization ( Fig. 2 ). Following intravenous SonoVue (2.5 mL), CH-EUS demonstrated: (1) the arterial phase—rapid hyperenhancement of periportal hepatic arterial branches with complete bile duct avascularity and (2) the portal venous phase—intense homogeneous portal vein enhancement versus persistent non-enhanced bile duct lumen, creating striking contrast ( Fig. 3 ). CH-EUS signals remained stable throughout the cardiac cycle. Under real-time CH-EUS guidance, a 19-gauge needle was advanced into the avascular ductal lumen. Successful biliary cannulation was confirmed by bile aspiration and contrast injection. A 7Fr nasobiliary drain was deployed without complications ( Fig. 4 ). Jaundice resolved within 1 week. At a 6-month follow-up, the stent remained patent with no recurrent jaundice or cholangitis ( Video 1 ).

**Video 1** Contrast-harmonic endoscopic ultrasound-assisted endoscopic ultrasound-guided hepaticogastrostomy for minimally dilated intrahepatic ducts.


This case extends CH-EUS applications from intraluminal pathology assessment to periductal anatomic navigation for EUS-HGS guidance when conventional imaging fails.

Endoscopy_UCTN_Code_TTT_1AS_2AH
